# Repellent Activity of TRIG (N-N Diethyl Benzamide) against Man-Biting Mosquitoes

**DOI:** 10.1155/2018/9037616

**Published:** 2018-01-09

**Authors:** Shandala Msangi, Eliningaya Kweka, Aneth Mahande

**Affiliations:** Division of Livestock and Human Disease Vector Control, Tropical Pesticides Research Institute (TPRI), P.O. Box 3024, Arusha, Tanzania

## Abstract

A study was conducted to assess efficacy of a new repellent brand TRIG (15% N-N Diethyl Benzamide) when compared to DEET (20% N-N Methyl Toluamide). The repellents were tested in laboratory and field. In the laboratory, the repellence was tested on human volunteers, by exposing their repellent-treated arms on starved mosquitoes in cages for 3 minutes at hourly intervals, while counting the landing and probing attempts.* Anopheles gambiae* and* Aedes aegypti* mosquitoes were used. Field evaluation was conducted by Human Landing Catch technique. During the night, the repellents were applied on arms and legs and mosquitoes landing on these areas were collected. In laboratory tests, TRIG provided complete protection (100%) against* Anopheles gambiae* when applied at 1.25 g, while DEET provided this at 0.75 g. When tested on* Aedes* aegypti, TRIG provided complete protection when applied at 1 g, compared to 0.5 g for DEET. In the field, when applied at a recommended dose, both TRIG and DEET achieved above 90% protection against both* An. arabiensis* and* Culex quinquefasciatus* and a Complete Protection Time of about 6 hrs against both species of mosquitoes. The performances of the two products were found to be comparable and TRIG was recommended for use as repellent against mosquito bites.

## 1. Introduction

While insect borne diseases are currently considered to be a major health problem in tropical and subtropical climate, mosquito borne diseases in particular are considered to be the cause of major human health problems in Sub-Saharan Africa [1]. Estimated two billion people are at risk from mosquito borne disease, mostly in tropics. In Africa alone, such diseases include, but are not limited to, malaria, yellow fever, filariasis, chikungunya, dengue, and haemorrhagic fever [2, 3] causing estimated 3 million deaths from malaria alone annually, mostly in Sub-Saharan Africa [2].

Several vector control measures, such as chemical, biological, environmental, and personal protection measures, are taken to prevent transmission of malaria and other mosquito borne diseases. Personal protection is one of the established methods to prevent mosquito bites. The use of mosquito repellents by individuals and communities plays an important role in reducing man-mosquito contact and preventing mosquito biting, consequently lowering down the disease risk [4]. In fact, in many circumstances, applying repellents to the skin may be the only feasible way to protect against insect bites [5]. Repellents act by disrupting host seeking and feeding of mosquitoes and therefore they represent an important tool in the fight against mosquito borne-diseases.

Efficiency of such existing personal protection tools against malaria vectors needs to be improved to suit various settings. Previous studies have shown repellents to have significant protection efficiency against disease vectors [6–9]. In view of increasing reports of insecticide resistance of mosquito vectors [10–12] more compounds need to be screened to develop more new effective repellents to complement the existing personal protection tools, like Long-Lasting Insecticidal Nets (LLIN) and Indoor Residual Sprays (IRS), as there is no single method of mosquito control that will prove completely effective in areas with high transmission [13].

We report results of a study conducted to evaluate biological efficacy of a new brand of mosquito repellent formulation against man-biting mosquitoes, both in laboratory and in the field.

## 2. Materials and Methods

### 2.1. Study Areas

The study was conducted during April and May, 2017. The laboratory studies were conducted at the Tropical Pesticides Research Institute laboratories in Arusha, Northern Tanzania. The field trials were conducted at Lower Moshi Field Station, 10 km South of Moshi town, North-eastern Tanzania (37°20′E, 3°21′S, 700 m above sea level). Paddy growing in irrigated land is the main activity and conducted throughout the year therefore providing mosquito breeding environment all year round.

### 2.2. Test Products

TRIG is a new brand of mosquito repellent petroleum jelly, with 15% N-N DIETHYL Benzamide, and was evaluated for its efficacy against man-biting mosquitoes. TRIG was supplied by Chemi & Cotex industries of Dar es Salaam Tanzania. DEET, a product of US ALDRICH, a repellent with 97% N-N Methyl Toluamide, was diluted to 20% and tested alongside TRIG as a gold standard for comparison purposes.

### 2.3. Test Mosquitoes

Two species of mosquitoes were used.* Anopheles gambiae* sensu stricto (Kisumu strain) and* Aedes aegypti* maintained in the laboratory at the Tropical Pesticides Research Institute (TPRI) insectaries for several decades were used for the study. The insectary is maintained at a temperature of 27 ± 2°C and 80 ± 6 relative humidity. Four- to six-day old female mosquitoes were prepared for the test by being starved overnight but provided with sugar solution before being starved. For each test, two hundred (200) such mosquitoes were contained in a metal frame cages (35–40 cm) ready for testing.

### 2.4. Laboratory Tests

Laboratory tests were conducted to estimate the effective dose as well as the Complete Protection Time of the products. The experiments were conducted following WHO guidelines [14] for efficacy testing of mosquito repellents for human skin.

Different weights of TRIG petroleum jelly were prepared and applied to 600 cm^2^ of the forearm, wrist to elbow, of human volunteer. The remaining hand area was covered by gloves in which mosquitoes could not bite. Four different weights were made, 0.50 g, 0.75 g, 1.0 g, and 1.25 g, and applied to one forearm. Ethanol absolute was used for diluting the 97% DEET to make 20% Ethanolic DEET and also applied to the arm in the similar way and same weight and used as a positive control. Ethanol absolute alone was used as a negative control on the second arm. The experiments were conducted in a room maintained at about 25°C and 75% relative humidity.

After application, the arms were left for 30 minutes to dry-up and then repellent-treated arm was inserted into the cage and exposed for 3 minutes, while counting the number of probing, followed by the negative control arm into the other cage. This procedure was done for both DEET and TRIG in different days, at 60-minute intervals (0, 1, 3, 4, and 5 hrs). The experiment was replicated three (3) times for each concentration. For each hour, the Complete Protection Time (CPT) was estimated as the average number of minutes elapsed between the time of inserting treated arm and the first mosquito probing attempt. The percentage protection (*P*) was estimated using the formula given under data analysis section bellow.

### 2.5. Field Trials

The field trials were conducted at Lower Moshi irrigation scheme area. Previous study [15] showed that* An. arabiensis *and* Culex quinquefasciatus* are the predominating mosquito species in the area. Six houses were randomly selected for the study, two each for TRIG, DEET, and control. Selected houses were 25–50 m apart. Six experienced mosquito collectors were assigned individually to the houses randomly. Two sets were made, each with three collectors, in which one applied DEET, the second TRIG, and the third Ethanol as a negative control. The products were applied on bare limbs, elbow to wrist (approx. 600 cm^2^) and knee to ankle (approx. 1300 cm^2^). Diluted DEET (20%) was applied at a rate of 1 ml on arms [14] and 2 mls on legs. TRIG was applied at the rate of 1.25 g on arms and 2.5 g on legs as per laboratory results. Applications of the products were made 30 minutes before commencement of mosquito collection to allow drying. The test was conducted from 19.00 hrs to 03.00 hrs by performing Human Landing Catch (HLC) in which all mosquitoes landing on the exposed limb of each volunteer were collected by aspiration, while recording the time for each first landing. The HLC was conducted outside the selected houses. The collected mosquitoes were separated on hourly basis and were transferred to holding paper-cups and later in the morning identified. To ensure that mosquito collectors were active throughout the collection period, they were requested to send a short message to the coordinator on every hour. In each of the two sets, both the position and treatment were rotated among the mosquito collectors daily for 9 consecutive days, in the form of 3 × 3 Latin square scheme. All volunteers were asked to observe any side effects of the products.

### 2.6. Data Analysis

The numbers of collected mosquitoes for each species were recorded daily, pooled for each of the 3 sets (2 houses for each treatment), log- transformed to normalise the data, and subjected to analysis of variance to compare their means among the three treatments. Complete Protection Time (CPT) was calculated as the average number of minutes elapsed between starting time and first mosquito landing. The percentage repellence (Protection) was estimated using the formula bellow.

(1) $$\begin{array}{c} P=\frac{\text{Number of mosquitoes collected in negative control}-\text{Number collected in repellent}}{\text{Number of mosquitoes collected in negative control}}\times 100. \end{array}$$

The means of percentage protection for DEET and TRIG was calculated as above and compared using Student's *t*-test.

### 2.7. Ethical Issues

To avoid exposure to unusual risk of malaria infection, the mosquito collectors were recruited from the same settings where the study was conducted. Additionally, they were given malaria prophylaxis from the nearby dispensary. The study participants, who were experienced mosquito collectors, were informed orally on the work and the intention for their participation and then given specially prepared forms to give their signed informed consent. The written and signed consent forms were then used to seek ethical clearance from the ethical committee of the Tropical Pesticides Research Institute (TPRI) before starting the work.

## 3. Results

Results for the efficacy evaluation of TRIG and DEET against* An. gambiae* and* Aedes aegypti* in laboratory are shown in Tables 1 and 2. Hundred percent (100%) protection against* An. gambiae *was achieved at 0.75 g by DEET while TRIG achieved this at 1.25 g. On* Ae. aegypti*, 100% protection was achieved by DEET at 0.5 g while it was achieved by TRIG at 1 g (Table 1). However, in all cases the differences between DEET and TRIG were found to be not significant (*P* > 0.05). Both species of mosquitoes were able to land immediately on Ethanol treated (control) arm when inserted into the cages and started to probe, Table 2. However, they took different period of time to be able to land on arms treated with different concentrations of the repellents. The highest Complete Protection Time (CPT) for TRIG against* An. gambiae* was recorded at 1.25 g while that for DEET was recorded at 0.75 g at which* An. gambiae* could not land for the 5 hrs of our observation. The highest Complete Protection Time (CPT) for TRIG on* Aedes* mosquitoes was achieved at 0.75 g while DEET was 0.5 g (Table 2).

Results of field trials on TRIG and DEET for the 9-day trial are shown in Table 3. The number of* An. arabiensis *and* Cx. quinquefasciatus* mosquitoes collected is shown in Table 3. Analysis of variance indicated no significant difference (*P* > 0.05) between the numbers of collected mosquitoes between DEET and TRIG. However both treatments showed significantly fewer (*P* < 0.05) mosquitoes collected than the control. Both TRIG and DEET achieved about 90% and above percentage protection against* An. arabiensis* and* Cx. quinquefasciatus* and a CPT of about 6 hrs against both mosquito species. Although DEET performed better than TRIG in many aspects, *t*-test comparison of percentage mean protection between the two indicated that the difference is not significant (*P* > 0.05). There were no recorded complaints on side effects of the products.

## 4. Discussion

TRIG was tested in both laboratory and field settings. In our laboratory tests, TRIG gave a Complete Protection Time (CPT) of 5 hrs and 100% protection against* An. gambiae* when applied at 1.25 g (2 mg/cm^2^). This rate was also used in the field trial, giving CPT of about 6 hrs and over 90% protection for both* An. arabiensis *and* Cx. quinquefasciatus* species. The rate was found to be almost the same as the “generous application” recommended by the manufacturer, which was estimated to be between 1 and 1.5 g. Although a different species of* Anopheles gambiae* complex was tested in the field, our results suggest that the product could be effective in both species. Performance of a similar product (N,N diethyl Benzamide) in a different formulation was also reported elsewhere [4] in India, in which, when applied at 10 mg/cm^2^, the percentage protection was 100% against Anopheline with average protection time of 11 hrs and it was 98.8% against* Cx. quinquefasciatus*, with average protection time of 9 hrs.

Elsewhere, field studies show significant reduction of biting rates when various repellents were used for personal protection. Uzzan et al. [16] reported a significant biting protection on exposed human subjects when compared to control group in a double blinded randomized placebo controlled study, in which four repellents containing DEET,* para*–menthane-3,8-diol, and picaridin were applied and then biting rates were monitored for 9 hours in Senegal. However, further studies may be required to establish the actual effect of such reduction of biting rates in disease transmission. For instance, a study conducted in Pakistan [17] showed that a repellent soap containing DEET was highly successful in reducing* Plasmodium falciparum* cases when compared to control group.

DEET is probably the best and oldest repellent, which is used since the 1950s [4]. Since then, there have been efforts to get new products with similar or better performance [18, 19]. DEET is known to be very effective [20–23] but also with very low risk of serious side effects [24, 25]. It is the most widely marketed chemical-based insect repellent, being used worldwide since the 1950s [22]. This could explain its use as a gold standard during evaluation of other new repellents against mosquitoes [14]. This high performance of DEET in terms of CPT and percentage (%) protection was also observed in our study. However, despite all these qualities, DEET has some setbacks. It may be washed off by perspiration or rain and its efficacy decreases significantly when outdoor temperatures rise [5, 26, 27].

In a comparative efficacy study of various repellents [22] authors had concluded that only products containing DEET offer long-lasting protection after a single application and that they can not recommend the use of any currently available non-DEET repellent to provide complete protection from arthropod bites for any sustained outdoor activity. However, in our study, the performance of TRIG, in terms of number of landing mosquitoes, Complete Protection Time, and percentage protection, was comparable to that of DEET. Although in many aspects DEET performed better than TRIG, the difference on their performance was statistically not significant.

Mosquito borne diseases remain a major threat to public health in Sub-Saharan Africa. While wide-scale mosquito control programs can reduce the risk of mosquito borne diseases to an acceptable level, its financial and operational requirements remain a major obstacle to its implementation. The use of personal protection strategies, particularly the use of various repellents, like TRIG, will therefore likely remain the most widely encouraged strategy to reduce mosquito borne disease risks [28]. TRIG is formulated as a petroleum jelly, a formulation which is normally used in cosmetics for long time and therefore likely to be accepted by many communities, as it will serve as both a cosmetic as well as a mosquito repellent.

## 5. Conclusions

We have deduced from this study that the evaluated product, TRIG (N-N Diethyl Benzamide) could play a significant role in reducing man-vector contact and thereby is likely to reduce the risk of malaria transmission. The products therefore supplement the already existing repellents.

## Figures and Tables

**Table 1:** Mean percentage protection of different concentrations of DEET and TRIG against *An. gambiae *and *Aedes aegypti* mosquitoes in laboratory tests.

Product concentration	Mean percentage (%) protection (±SE)			
	*Anopheles gambiae*		*Aedes aegypti*	
	DEET	TRIG	DEET	TRIG
0.50 g	95.8 ± 4.5	95 ± 4.7	100 ± 0	99.7 ± 0.6
0.75 g	100 ± 0	95.3 ± 8	100 ± 0	99.8 ± 0.6
1.00 g	100 ± 0	96.5 ± 8	100 ± 0	100 ± 0
1.25 g	100 ± 0	100 ± 0	100 ± 0	100 ± 0

**Table 2:** Complete Protection Time (CPT) in hours for DEET and TRIG against mosquito bites in laboratory tests.

Product concentration (g)	*Anopheles gambiae*			*Aedes aegypti*		
	DEET (CPT)	TRIG (CPT)	Control (CPT)	DEET (CPT)	TRIG (CPT)	Control (CPT)
0.50 g	0	0	0	5 hrs	4 hrs	0
0.75 g	5 hrs	2 hrs	0	5 hrs	5 hrs	0
1.00 g	5 hrs	4 hrs	0	5 hrs	5 hrs	0
1.25 g	5 hrs	5 hrs	0	5 hrs	5 hrs	0

**Table 3:** Mean percentage (%) protection and Complete Protection Time of DEET and TRIG against mosquitoes in the field trials.

Mosquito species collected	Treatment	Total mosquito collected	Mean% protection (±SE)	Complete Protection Time in Hrs (±SE)
*An. arabiensis*	DEET	9	94.7 ± 4.4	7 ± 1.6
	TRIG	19	89 ± 14	6.3 ± 1.7
	CONTROL	172		

*Cx. quinquefasciatus*	DEET	51	91.3 ± 12.2	6.2 ± 1.4
	TRIG	38	93.5 ± 5.2	5.8 ± 1.6
	CONTROL	590		
